# Dynamic Bayesian network in infectious diseases surveillance: a simulation study

**DOI:** 10.1038/s41598-019-46737-0

**Published:** 2019-07-17

**Authors:** Tao Zhang, Yue Ma, Xiong Xiao, Yun Lin, Xingyu Zhang, Fei Yin, Xiaosong Li

**Affiliations:** 1 Department of Epidemiology and Health Statistics, West China School of Public Health and West China Fourth Hospital, Sichuan University, Sichuan, China; 2 Department of Systems, Populations and Leadership, University of Michigan, School of Nursing, Ann Arbor, USA

**Keywords:** Infectious diseases, Risk factors

## Abstract

The surveillance of infectious diseases relies on the identification of dynamic relations between the infectious diseases and corresponding influencing factors. However, the identification task confronts with two practical challenges: *small sample size* and *delayed effect*. To overcome both challenges to imporve the identification results, this study evaluated the performance of dynamic Bayesian network(DBN) in infectious diseases surveillance. Specifically, the evaluation was conducted by two simulations. The first simulation was to evaluate the performance of DBN by comparing it with the Granger causality test and the least absolute shrinkage and selection operator (LASSO) method; and the second simulation was to assess how the DBN could improve the forecasting ability of infectious diseases. In order to make both simulations close to the real-world situation as much as possible, their simulation scenarios were adapted from real-world studies, and practical issues such as *nonlinearity* and *nuisance variables* were also considered. The main simulation results were: ① When the sample size was large (*n* = 340), the true positive rates (TPRs) of DBN (≥98%) were slightly higher than those of the Granger causality method and approximately the same as those of the LASSO method; the false positive rates (FPRs) of DBN were averagely 46% less than those of the Granger causality test, and 22% less than those of the LASSO method. ② When the sample size was small, the main problem was low TPR, which would be further aggravated by the issues of *nonlinearity* and *nuisance variables*. In the worst situation (i.e., small sample size, nonlinearity and existence of nuisance variables), the TPR of DBN declined to 43.30%. However, it was worth noting that such decline could also be found in the corresponding results of Granger causality test and LASSO method. ③ Sample size was important for identifying the dynamic relations among multiple variables, in this case, at least three years of weekly historical data were needed to guarantee the quality of infectious diseases surveillance. ④ DBN could improve the foresting results through reducing forecasting errors by 7%. According to the above results, DBN is recommended to improve the quality of infectious diseases surveillance.

## Introduction

The profiles of infectious diseases epidemics are influenced and shaped by many exogenous variables related to weather, environment, economy, social customs, and so on^1–4^. These exogenous variables, if appropriately utilized, would be extremely helpful for the surveillance of infectious diseases^5–7^. For example, Earnest *et al*.^5^ found that weekly average temperature, average relative humidity and El Niño Southern Oscillation Index (SOI) were significantly and independently associated with dengue notifications. It is then natural to come up with the idea that such exogenous variables could further be incorporated in the infectious diseases surveillance system to monitor the epidemics in a prospective way, so that once the exogenous variables have changed (such as climate change^7^), the surveillance system could release timely alert on infectious diseases. If the alert is accurate and timely, then proper prevention measures could be taken to avoid the potential enormous losses of properties and lives. To fulfil this profound mission, the fundamental point is to identify the *dynamic relations*, which means getting to know the time-lag effect of historical exogenous variables on the current or future epidemics of infectious disease (e.g., the influence of temperature change in the last week on the current epidemics of influenza).However, due to the complexity of real world, this identification task always confronts with great challenges. Although many challenges may only be restricted to some certain types of infectious diseases, there still exist two major ones: *small sample size* and *delayed effect*.

### **Small Sample Size**

In a real-world situation, especially for emerging and re-emerging infectious diseases, urgent health-policy decision is usually required even though there is only limited amount of data at hand, which leads to the *small sample size* challenge. This challenge would in turn cause the lack of statistical power and large standard errors, and consequently decrease the validity and precision of surveillance analysis^8^.

### Delayed Effect

It emphasizes the temporal characteristics of the dynamic relations which need to be identified. Since the delayed effect takes temporal information into account, it differs from the static effect, which represents a snapshot of the underlying relations at a particular moment in time and makes no use of temporal information. However, even the static effect is sometimes hard to be identified, especially when the number of variables increases^9^. Therefore, the delayed effect will undoubtedly become much harder for identification than the static effect due to the extra temporal information.

To overcome the challenges in the identification of dynamic relations, previous researches have proposed dynamic Bayesian network (DBN) as a promising approach. For example, Lèbre^10^ has shown that under some mild assumptions, the joint distribution of multivariate time series could be reliably represented as a DBN. Furthermore, the work of Zou and Feng^11^ proposed a comparative study between the DBN and Granger causality test on both synthesized and experimental data in genomics, which suggested that when the sample size was small, the DBN could outperform the Granger causality test in terms of validity (i.e., true positive rate and false positive rate). All these good properties of DBN also extend its applications to other fields outside genomics. Recently, a few studies are beginning to apply DBN to the surveillance of infectious diseases. For example, Sebastiani *et al*.^12^ used DBN to integrate different sources of data to improve the surveillance of influenza. Lau and Smith^13^ demonstrated the use of Bayesian network with a leptospirosis example. All the works indicated the potential values for developing dynamic tools based on DBN to improve public health decision and intervention.

Although much work of DBN has been made, previous studies seldom directly considered verifying whether DBN could indeed overcome the aforementioned challenges (*small sample size* and *delayed effect*) of infectious diseases surveillance. To our knowledge, such consideration was necessary for at least two reasons mentioned below.

1. Data availability varies dramatically from one discipline to another, thus leading to different meanings of *small sample size* and *delayed effect* across different disciplines. For example, DBN has been successfully applied to identify effective connectivity in human brain from the functional magnetic resonance imaging (fMRI)^14^. On the one hand, the fMRI data and the infectious diseases surveillance data have some common structural characteristics of time series data such as *autocorrelation* (the correlation between the current observation and its historical records) and *periodicity* (the data exhibit repetitive or regular behaviours over time)^15^, which suggests the applicability of DBN from fMRI to infectious diseases surveillance. However, on the other hand, their differences are also obvious: in the fMRI study, the data acquisition intervals could be accurate to seconds, which means that it only takes quite a few times to collect a large amount of fMRI data (e.g., 900 observations of data could be obtained within 10 minutes^14^). Instead, the frequency of data collection in infectious diseases surveillance is often by day or by week, meaning that months or even years are needed to collect hundreds of observations. Therefore, the scales of sample size and time-delay are quite different between fMRI and infectious diseases surveillance. In other words, 900 observations is typically large sample size in the situation of infectious diseases surveillance^16,17^, but small in fMRI study; and collecting data by hour may indicate short time-delay in infectious diseases surveillance, but long time-delay in fMRI study. Such differences remind us that the success of DBN in other disciplines (e.g., fMRI) should not be treated as a guarantee of its successful application to infectious diseases surveillance. On the contrary, due to discipline differences, it is still necessary to verify the performance of DBN when dealing with *small sample size* and *delayed effect* challenges in context of infectious diseases surveillance.
2. Besides the challenges of *small sample size* and *delayed effect*, the surveillance of infectious diseases also confronts with other issues, such as *nonlinearity* and *nuisance variables* issues. The *nonlinearity* means the nonlinear influencing mechanism of exogenous variables on the infectious diseases^18^. It could make the data structure more complicated, and increase the difficulty of dynamic relation identification. The *nuisance variables* issue means that due to the lack of proper methods for identifying dynamic relations, some collected variables may actually have nothing to do with the infectious diseases of interest. From the view of statistical analysis, the nuisance variables could not only increase the difficulty of analyzing, but also deteriorate the validity and precision of the analysis results. Therefore, the verification work of DBN in infectious diseases surveillance would be more convincing if *nonlinearity* and *nuisance* issues are considered.

To this end, this study uses simulation approaches to verify how DBN could deal with the *small sample size* and *delayed effect* challenges in infectious diseases surveillance. Meanwhile, the *nonlinearity* and *nuisance* issues are also considered to some extent. The remaining paper is organized as follows: In Section 2, we present the conceptual framework of the DBN, as well as a brief description of other approaches for model comparison. Furthermore, Sections 3 and 4 demonstrate the application of DBN in infectious diseases surveillance with two simulations: one is to evaluate the performance of DBN, and the other one is to show how the DBN could help to improve the forecasting ability of infectious diseases. The simulation scenarios of both studies are adapted from real-world studies to enhance their practical sense. Finally, Section 5 ends the paper with a discussion.

## The Method

### Dynamic bayesian network

Let ***X***_***t***_ = $({X}_{t}^{0},{X}_{t}^{1},\ldots ,{X}_{t}^{m})^{\prime}$ be (*m* + 1)-dimension time series observed at time *t* (*t* = 1, 2, 3, …). For example, let $\,{X}_{t}^{0}$ be the incidence of infectious disease and ${X}_{t}^{i}$ (*i* = 1, 2, …, *m*) the *m* exogenous variables contained in the surveillance data system. The DBN is a special case of a diagrammatic representation of probability distributions^19^. It uses nodes to represent the variables and arcs to represent the dynamic relations between any pair of variables at successive time points based on the past observations. According to the theory of Opgenrhein and Strimmer^20^, the DBN could be learned from the vector autoregressive (VAR) model with an effective model selection procedure. This learning process involves three steps.

1. Building VAR model based on the time series data {***X***_***t***_} (*t* = 1, 2, 3, …). The VAR model is an extension of traditional autoregressive (AR) model. For example, the influence of the last *p* historical data on the current observations could be characterized by VAR(*p*) model as below.
  1 $${{\boldsymbol{X}}}_{t}={\mu }_{t}+{{\boldsymbol{\varphi }}}_{1}^{\ast }{{\boldsymbol{X}}}_{t-1}+\cdots +{{\boldsymbol{\varphi }}}_{p}^{\ast }{{\boldsymbol{X}}}_{t-p}+{{\boldsymbol{a}}}_{t}.$$
  In model (1), ***µ***_***t***_ = $({\mu }_{t}^{0},{\mu }_{t}^{1},\ldots ,{\mu }_{t}^{m})^{\prime}$ is a (*m* + 1)-dimension constant vector and ${{\boldsymbol{\varphi }}}_{{\boldsymbol{i}}}^{\ast }$ = {${{\Phi }}_{i}^{(j,k)\ast }$} (*i* = 1, 2, …, *p*; *j* = 0, 1, …, *m*; *k* = 0, 1, …, *m*) are (*m* + 1) × (*m* + 1) matrices, and ***a***_*t*_ = $({a}_{t}^{0},{a}_{t}^{1},\ldots ,{a}_{t}^{m})^{\prime}$ is a sequence of independent and identically distributed random vectors with mean zero and constant covariance matrix. The unknown parameters in VAR model could be initially estimated through the least squared method. For a better understanding of model (1), the ${{\boldsymbol{\varphi }}}_{{\boldsymbol{i}}}^{\ast }$ could be interpreted as the lag-*i* (*i* = 1, 2, 3, …) autoregressive coefficient matrix, which measures the dynamic dependencies between ***X***_***t***_ and ***X***_***t***-***i***_. Consequently, there are (*m* + 1) equations in model (1), and the first one is
  2 $${X}_{t}^{0}={\mu }_{t}^{0}+{\sum }_{i=1}^{p}{\sum }_{j=0}^{m}{{\rm{\Phi }}}_{i}^{(j,0)\ast }{X}_{t-i}^{j}+{a}_{t}^{0},$$
  which regresses ${X}_{t}^{0}$ (i.e., the incidence of an infectious disease at time *t*) on its own previous observations as well as past observations of other *m* exogenous variables. Besides, the other *m* equations in model (1) construct the regression relations among the *m* exogenous variables.
2. Using the James-Stein shrinkage approach to improve the estimated coefficients of Eq. (1) in the first step. It is well known that the least squared estimates are highly depended on the empirical covariance matrix of ***X***_***t***_ (defined as ***S***), hence such estimates may run into problems when ***S*** is inefficient and ill-conditioned, especially when there are a large number of exogenous variables (i.e., *m* is very high). The James-Stein shrinkage approach could overcome such problem by first replacing *S* with shrinkage covariance S* and consequently estimating regression coefficients through S*. In such a way the James-Stein shrinkage approach would shrink some trivial coefficients in ${{\boldsymbol{\varphi }}}_{{\boldsymbol{i}}}^{\ast }$(*i* = 1, 2, …, *p*) to be zero so that the remaining non-zero coefficients can be more indicative of potentially important dynamic relations^20^.
3. Once the estimated coefficients of the vector autoregressive model are improved, they could then be used to label the relative importance of each relation. That is, the larger the improved coefficient is, the more important its corresponding relation is thought to be. Furthermore, the DBN uses arcs to represent those relatively important relations.

During this process, to identify significant dynamic relations while avoiding multiple comparisons problem, the local false discovery rate (lfdr) approach is implemented. The lfdr is the Bayes posterior probability that a hypothesis is null given its statistic *x*, i.e., lfdr(*x*) ≡ *Pr*(null|*x*). In addition, Efron^21^ suggests the significance threshold of lfdr to be 0.2, which yields *Pr*(non-null|*x*) four times higher than *Pr*(null|*x*) to balance type I and II errors.

The DBN could be implemented in R 3.2.3, a free software environment for statistical computing and graphics. Computing Packages {*bnlearn*}, {*GeneNet*} and {*MSBVAR*} are downloaded from the Comprehensive R Archive Network (CRAN) at http://cran.r-project.org/ and installed in advance.

### Other approaches for model comparison

To better evaluate the performance of DBN, two conventional approaches, i.e., the Granger causality test and LASSO method, also served as benchmarks for comparison.

#### Granger causality test

The Granger causality test implements all possible bivariate Granger causality tests for *m* variables^22^. It defines one time series $\{{X}_{t}^{i}\}$ as Grange-cause for another time series $\{{X}_{t}^{j}\}$ if the lagged values of $\{{X}_{t}^{i}\}$ provide statistically significant information for predicting the current value of $\{{X}_{t}^{j}\}$ (*i*, *j* = 0,1,…,*m*, *i* ≠ *j*). The null hypothesis is that the past *p* values of $\{{X}_{t}^{i}\}$ are of no use in predicting the value of $\{{X}_{t}^{j}\}$. The procedure of Granger causality test involves regressing $\{{X}_{t}^{j}\}$ on the *p* past values of $\{{X}_{t}^{i}\}$.Then an *F*-test by single equation ordinary least squared models is carried out to determine whether the coefficients of the past values of $\{{X}_{t}^{i}\}$ are zero. Similar to DBN, the Granger causality test also uses the local FDR approach to handle the multiple comparisons problem. The Granger causality test could be conducted in the *R* environment by the command *granger.test*.

#### The least absolute shrinkage and selection operator (LASSO) method

For a regression model with the current value of $\{{X}_{t}^{j}\}$ as the dependent variable and the other *m* series $\{{X}_{t}^{i}\}$(*i*, *j* = 0,1,…,*m*, *i* ≠ *j*) as the predictors, the LASSO method^23^ could compact the model by shrinking the estimated regression coefficients and setting a number of them to zero, thus identifying significant regression relations among data. The LASSO method is carried out by minimizing the sum of the mean squared error and the weighted *L*_1_ penalty. The weight of the *L*_1_ penalty is chosen via 10-fold cross-validation. A grid of candidate weights are selected to compute the cross-validation error. Then the weight value corresponding to the smallest cross-validation error is selected as the optimal weight. Finally, the model is re-fit using all available observations and the optimal weight. The LASSO could be conducted in the *R* environment by the command *lars* and *cv*.*lars*.

## Simulation 1: the Performance Evaluation of DBN

In order to assess the performance of DBN in the surveillance of infectious diseases, two specific aims were set in Simulation 1. One was to evaluate the performance of DBN in context of the challenges of *delayed effect* and *small sample size*. The other one was to compare the DBN with the Granger causality test and LASSO method. The following part described the simulation design and performance measures, and interpreted the results of Simulation 1.

### Simulation design

The simulation scenarios were set in four steps: the first step was to construct the structure of the simulation model, the second step was to further set the simulation scenarios according to the model structure. Then the parameters of the simulation model were determined in the third step. Finally, the last step generated the simulation data from the simulation model.

#### Structure construction

**Figure 1:**
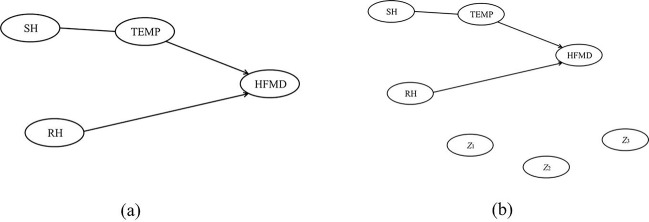
(**a**) The simulation structure of Simulation 1 in the absence of nuisance variables; (**b**) the simulation structure of Simulation 1 in the presence of nuisance variables.

To make the simulation close to the real-world surveillance as much as possible, the simulation scenarios were adapted from previous studies^24–26^ on the real-world surveillance data of hand, foot, and mouth disease (HFMD) in Beijing in 2009. HFMD is a common infectious disease caused by a group of enteroviruses such as Coxsakievirus A16 (CA16) and Enterovirus 71 (EV71), which is mainly transmitted by nasopharyngeal secretions such as saliva or nasal mucus^27^. Its epidemics can occur almost all year round except winter. In recent years, HFMD epidemics are frequent and widespread in the Asia-Pacific region^28^. For example, there are over 4.5 million cases reported in mainland China from January 2013 to December 2014. Besides, Kol *et al*.^29^ estimated that HFMD causes 96 900 (95% CI 40 600 to 259 000) age-weighted DALYs per annum in eight high-burden countries in East and Southeast Asia. Furthermore, given that previous studies suggest that the weather-HFMD relationship can be delayed because of the incubation period of infectious disease^25^, the simulation model sets the weekly cases of HFMD (*HFMD*) to be dynamically related with both the weekly average value of temperature (*TEMP*) and relative humidity (*RH*) one or two weeks ago. Meanwhile, the simulation model also sets contemporary relation between the weekly average value of sunshine hour (*SH*) and temperature. The above relations could be visualized as shown in Fig. 1(a), where the directed arcs indicate that the variable at the tail has a delayed effect on the variable at the head (i.e., *TEMP* → *HFMD*, *RH* → *HFMD*), the undirected arcs suggest that the two connected variable are contemporarily related (i.e., *SH*-*TEMP*), and the absence of arc between two variables means that they are not related. Of course there may be some other factors influencing the incidence of HFMD as well, but since this is not a specific study on how to prevent HFMD, it is not necessary to include all the possible influencing factors of HFMD. On the contrary, we selected the widely accepted factors (temperature and humidity) to illustrate that the results of our study could coincide with common knowledge and make practical sense.

Futhermore, the structure of Fig. 1(a) could be translated into mathematical form as in Eq. (3),

3 $$\{\begin{array}{c}TEM{P}_{t}={\alpha }_{1}+{\beta }_{1}\ast {f}_{1}(TEM{P}_{t-1})+{\beta }_{2}\ast \,\sin [2{\rm{\pi }}(t-13)/52]+{\varepsilon }_{t,1}\,\\ S{H}_{t}={\alpha }_{2}+{\beta }_{3}\ast {f}_{2}(S{H}_{t-1})+{\beta }_{4}\ast {f}_{3}(TEM{P}_{t})+{\varepsilon }_{t,2}\\ (1-{B}^{52})R{H}_{t}=(1-{\alpha }_{3}\ast B){\varepsilon }_{t,3}\,\\ HFM{D}_{t}={\alpha }_{4}+{\beta }_{5}\ast {f}_{4}(HFM{D}_{t-1})+{\beta }_{6}\ast {f}_{5}(HFM{D}_{t-2})+{\beta }_{7}\ast {f}_{6}(TEM{P}_{t-1})+{\beta }_{8}\ast {f}_{7}(R{H}_{t-1})+{\varepsilon }_{t,4}\end{array}$$

where *f*_*i*_() (*i* = 1, 2, …, 7) represented some kinds of functional transformation on the orginal data, which would be further considered in the following steps. The arcs in Fig. 1(a) was reflected by the regression relations between dependent and independent variables. Since weekly data was used in the prototype studies of Simualtion 1, the time slice *t* was also defined by week. A sine function sin[2*π*(*t* − 13)/52] was added to represent the periodic trend of temperature. In addition, relative humidity was charaterized by seasonal autoregressive time series model because of its seasonality and the absence of assumed influence of other variables upon it.

Both Fig. 1(a) and Eq. (3) showed the structure of simulation model, that was, how the variables of interest were dynamically related with each other. Furthermore, the parameters of Eq. (3) (*α*_1_ to *α*_4_, *β*_1_ to *β*_8_) needed to be determined before simulated data could be generated from the simulation model.

#### Scenario setting

According to the aims of Simulation 1, there were eight scenarios (Table 1), which consisted of sample size, mechanism and existence of nuisance variables. Considerations for scenarios setting were given as below.

**Table 1:** The settings of the simulation scenarios.

No. of Simulation	Sample Size	Mechanism	Existence of Nuisance Variables
1	340	linear	N
2	52	linear	N
3	340	nonlinear	N
4	340	linear	Y
5	52	nonlinear	N
6	52	linear	Y
7	340	nonlinear	Y
8	52	nonlinear	Y

Small sample size *versus* large sample size: Since the simulation scenarios imitated the weekly HFMD and meteorological factors, it was plausible to set the small sample size scenario as *n* = 52, which meant the researcher only got one single year’s data at hand. On the contrary, the large sample size scenario was set to be *n* = 340, which suggested the availability of more than six years’ data. Furthermore, because the *sample size* challenge was one of the most concerned problems in surveillance practice, we also carried out simulations in cases where *n* = 104 (two years), 156 (three years), 208 (four years) and 260 (five years), respectively. Thus, the simulation results would serve as a reference for other researchers to choose the appropriate sample size in their studies as well.

Delayed effect: The delayed effect was demonstrated by both the directed arcs in Fig. 1(a) and the time-lag variables (i.e., *TEMP*_*t*−1_, *SH*_*t*−1_, *HFMD*_*t*−1_ and *HFMD*_*t*−2_) in Eq. (3).

Linearity *versus* nonlinearity: The linear mechanism set the function *f*_*i*_ () (*i* = 1, 2, …, 7) in Eq. (3) to be the input variable itself. As for the nonlinear mechanism, nonlinearity could indeed vary in tremendous ways, but due to the limited aims of this study (nonlinearity was only considered as a concurrent issue rather than the major aim of this study), we chose the sigmoid function as a special type of nonlinearity. In addition, because the sigmoid function was widely used in nonlinear models (such as neural network^30^), it was a typical representative of nonlinear cases.

Presence *versus* absence of nuisance variables: The *nuisance variables* issue was considered by adding three nuisance variables (*Z*_1*,t*_, *Z*_2*,t*_, *Z*_3*,t*_) into simulation. As shown in Fig. 1(b), *Z*_1*,t*_, *Z*_2*,t*_ and *Z*_3*,t*_ were set to be independently distributed, and each of them was set to represent a typical form of nuisance variables: *Z*_1*,t*_ was the form whose current observation was only influenced by its previous ones (e.g., the variable irrelevant to infectious disease and its influencing factors); *Z*_2*,t*_ was the form which was caused by errors (e.g., the measurement error during data collection); and *Z*_3*,t*_ was the form which was influenced by both its previous values and errors (e.g., the variable was irrelevant to the surveillance data, but influenced by the measurement error during data collection). Furthermore, because the above features of *Z*_1*,t*_, *Z*_2*,t*_ and *Z*_3*,t*_ just corresponded separately with the definitions of three commonly-used time series models^31–33^, i.e., the autoregressive (AR) model, moving average (MA) model and autoregressive moving average (ARMA) model, it was reasonable to characterize *Z*_1*,t*_, *Z*_2*,t*_ and *Z*_3*,t*_ by those three time series models, respectively. Since the three models have good properties in theory and great successes in application, they could guarantee the representativeness of nuisance variables in reality to some extent.

#### Parameter determination

The second step involved determining the parameters in the simulation model of Eq. (3). To assure the simulation maintained the key characteristics of surveillance data, we estimated the parameters in Eq. (3) (i.e., *α*_1_ to *α*_4_, *β*_1_ to *β*_8_) by fitting the models to the real dataset of HFMD and meteorological factors of Beijing in 2009.

#### Data generation

Once the function *f*_*i*_ () (*i* = 1, 2, …, 7) and parameters in Eq. (3) were determined, Eq. (3) could be used to generate the simulation data. For each scenario in Table 1, the data generation process was repeated 5000 times, leading to 5000 replicates. For each replicate, the initial values of *HFMD*, *SH*, *RH* and *TEMP* were randomly selected from standard normal distribution, then the initial values were put into Eq. (3) to forecast the new values of *HFMD*, *SH*, *RH* and *TEMP* in the next time point, and again the newly forecasted values were put into Eq. (3) to make another new round of forecasts, and so forth. In such an iterative way, the simulation data could be generated. In addition, the length of each replicate was (500 + *n*), where *n* was the sample size listed in Table 1. To assure the steady of data generation process, the first 500 time points of each replicate were discarded as a burn-in, therefore the left *n* time points in each replicate were used for the evaluation of model performance.

### Performance measures

The performance of DBN could be evaluated by applying it to the simulated data. For each replicate, DBN would identify some of the variables to be dynamically related with each other. Then by comparing the DBN-identified dynamic relation with the true model structure in Fig. 1(a) or (b), it could be known whether the DBN-identified dynamic relation truly existed or not. In other words, once DBN identified a dynamic relation between two variables, it may be either *true positive* (the truly existed dynamic relation between two variables being successfully identified) or *false positive* (the truly non-existed dynamic relation being falsely identified). Since there were 5000 replicates for each scenario in Table 1, two performance measures were taken: true positive rate (TPR) and false positive rate (FPR). For each truly existed dynamic relation in Fig. 1(a) or (b), its TPR (also known as sensitivity) was defined as the proportion of the 5000 replicates when it was successfully identified by DBN. On the contrary, for each truly non-existed dynamic relation in Fig. 1(a) or (b), its FPR referred to the proportion of the 5000 replicates when it was falsely identified by DBN. Of course, the TPR and FPR of the LASSO method as well as Granger causality test could be defined in the similar way. Since both TPR and FPR were well-defined measures of classification test, they would also be adequate for evaluating and comparing performances of DBN, LASSO and Granger causality test in this study.

### Results interpretation

**Figure 2:**
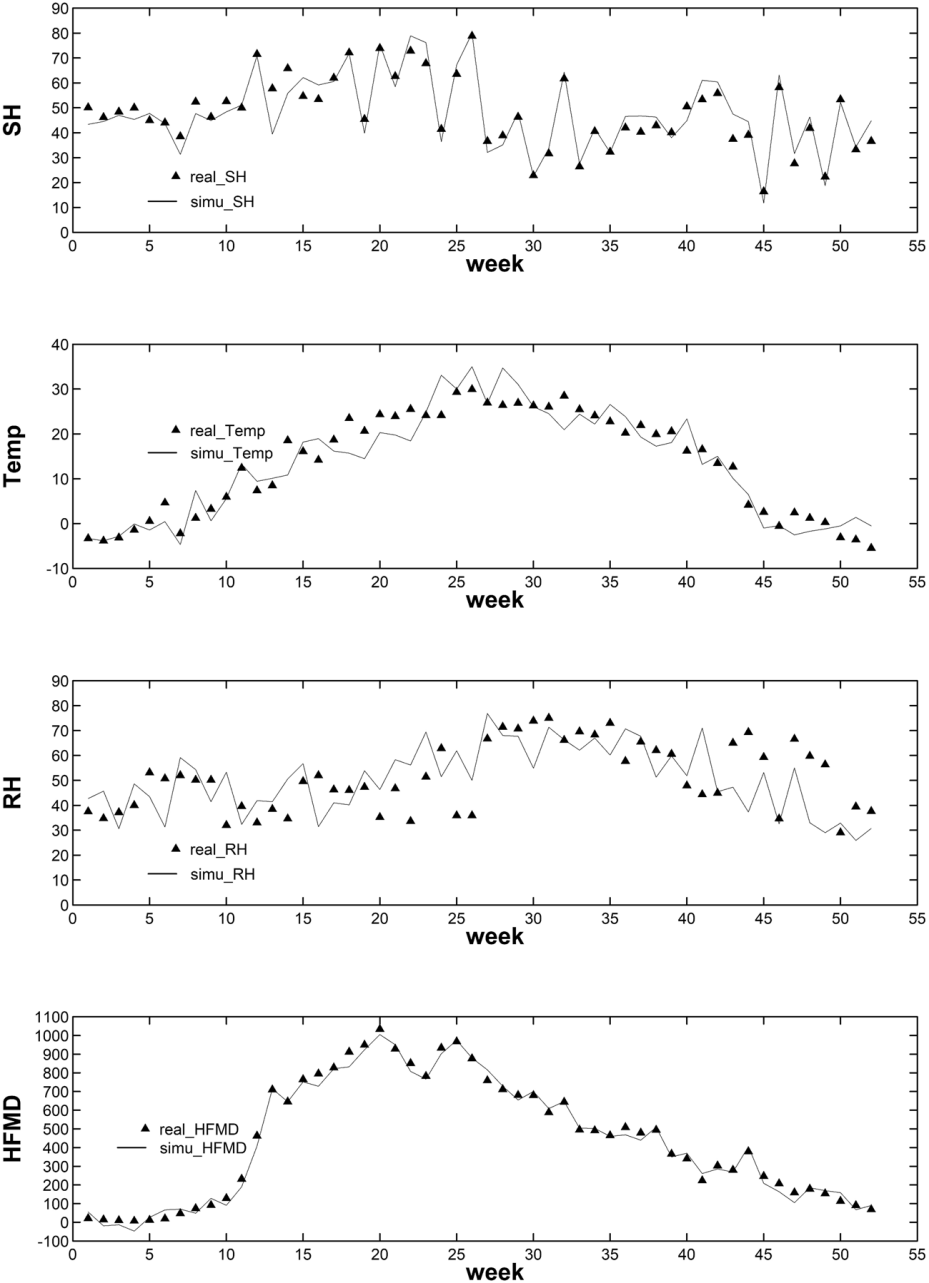
The time plots of the real and simulated data sets

The simulated data was in consistent with the real data. Figure 2 showed that the real and simulated data sets matched temporally. Besides, Table 2 listed the comparison of variables in the simulated and real data set of Beijing in 2009. The paired-sample *t*-test or the Wilcoxon signed rank test was utilized for comparison. It thereby suggested that the simulated time series basically imitated the real ones and did make practical sense. Furthermore, the results of model comparison were reported below.

**Table 2:** The comparison between the real and simulated data.

Variable	Real Data		Simulated Data		Test Statistics*	*P* value
	Mean	std	Mean	std		
Sunshine	47.97	14.18	48.13	15.25	*t* = −0.2198	0.8269
Temperature	13.37	11.46	13.14	11.79	Z = −0.4243	0.6714
RH	51.15	13.53	50.42	13.38	*t* = 0.3861	0.7010
HFMD	446	324.60	439.16	321.52	Z = −0.1387	0.8897

*t stands for the *t* statistics of the paired-sample *t*-test, and *Z* stands for the *Z* statistics of the Wilcoxon signed rank test for paired data.

#### Model comparison

**Figure 3:**
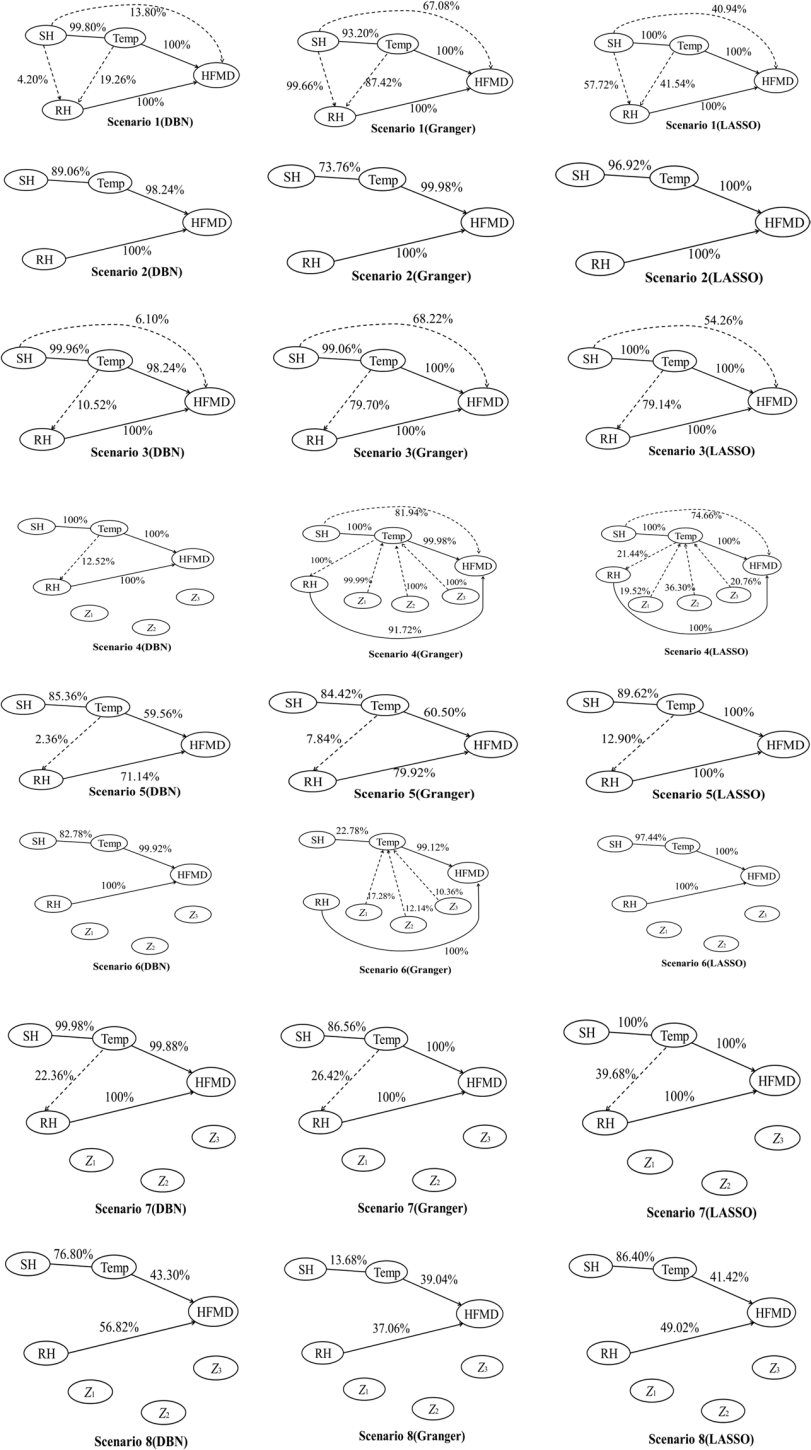
The results of dynamic Bayesian network (DBN), Granger causality test and LASSO method applied on each scenario, where the solid lines represented the true positive rate (TPR), and dashed lines represented the false positive rate (FPR).

Figure 3 demonstrated the results of DBN, Granger causality test and LASSO method, where the solid line represented the TPR, the dashed line represented the FPR, and the numbers near lines were the corresponding values of TPR or FPR. Meanwhile, rates less than 1% were omitted in Fig. 3 for clarity.

Since the performance of each approach varied dramatically with different sample sizes (*n* = 52 or *n* = 340), the results could be separately summarized as below.

- The comparison results when sample size was large (*n* = 340).
  When the sample size was large (Scenario 1,3,4 and 7), all the TPRs of DBN were greater than 98%, which were slightly higher than those of the Granger causality test and approximately the same as those of the LASSO method. Furthermore, the average FPR of DBN was 46% less than that of the Granger causality test, and 22% less than that of the LASSO method. Hence, it suggested that when the sample size was large, the DBN performed better than the other two approaches, especially in terms of FPR.
- The comparison results when sample size was small (*n* = 52).

When the sample size was small (Scenario 2, 5, 6 and 8), the main problem was low TPR due to the lack of test power, which was further aggravated by the issues of nonlinearity and the existence of nuisance variables. For example, in Scenario 2 (*n* = 52, linear relation and no nuisance variable), the TPRs of DBN ranged from 89.06% to 100%. However, the performance of DBN was getting worse and worse as nonlinearity and nuisance variables were involved. In the worst situation (i.e., Scenario 8), the TPR of DBN declined to as low as 43.30%. Such a decline could also be found in the corresponding results of Granger causality test and LASSO method. Over the four simulation scenarios of small sample size, the average TPR of DBN was 80.25%, which was 13% higher than that of the Granger causality test, but 8% less than that of the LASSO method. This suggested that the DBN was not as powerful as the LASSO method to identify dynamic relations when the sample size was small. But it should also be noted that in Scenario 8, the lowest TPR of LASSO method (41.42%) was even lower than that of the DBN (43.30%). Since all these three approaches performed poorly in such situation with very low TPRs, it was meaningless to select a good one from all these poor candidates.

Moreover, the comparison of FPR could also provide some indications, albeit it was not the main problem in small sample size issue. In Scenario 5, the FPR of the DBN was 2.36% (from *Temp* to *RH*), which was less than the corresponding rate of Granger causality test (7.84%) and LASSO method (12.90%). This coincided with the conclusion of Opgenrhein and Strimmer^20^, which suggested the DBN performed better than the LASSO method with lower false positive rates especially when the sample size was small (between 5 and 200). In addition, the promising feature was that there were 100 nodes and 200 edges in the study of Opgenrhein and Strimmer, much larger than those in this study. Therefore, it indicated that the DBN may be an optimal choice for infectious diseases surveillance if more and more variables could be included.

#### Sample size issue

As mentioned above, the sample size issue played an important role in determining the performance of DBN in terms of TPRs. If we classified those eight scenarios by mechanism and existence of nuisance variables (i.e., Scenario 1 *versus* 2, Scenario 3 *versus* 5, Scenario 4 *versus* 6, and Scenario 7 *versus* 8.), it could be concluded that the TPRs of DBN were always higher in cases of large sample size (*n* = 340) than small sample size (*n* = 52). Of course, these results should not be mistakenly interpreted as that *delayed effect*, *nonlinearity* and *nuisance variables* were not important; instead, it indicated that when the sample size was large enough, the DBN still remained robust to those three challenges.

**Figure 4:**
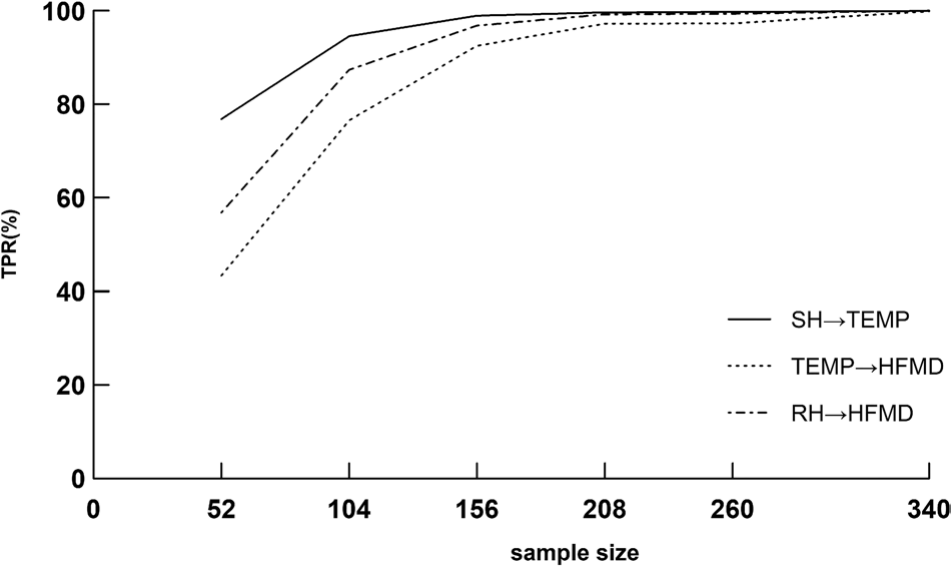
The curve of sample size and TPR (%).

To better illustrate the influence of sample size on DBN’s performance, we also carried out another four additional simulation scenarios in context of nonlinearity and nuisance variables, with the sample size being 104(two years), 156(three years), 208(four years) and 260 (five years), respectively. The reason for such scenario setting was to establish the advisable sample size for the application of DBN in infectious diseases surveillance under a situation close to the real-world situation as much as possible. In addition, the TPR was taken as the performance measure, since it has just been shown in this study that lower TPR was the main problem of validity when the sample size was insufficient. Figure 4 illustrated that the TPR of DBN increased as the sample size became larger and larger. Specifically, when the sample size came to 156, the TPRs turned out to be acceptable (varying from 92.48% to 98.92%). Therefore, it suggested that at least three years of weekly historical data were needed for the use of DBN in infectious disease surveillance.

## Simulation 2: How could the DBN Improve the Forecasting of Infectious Diseases in Practice?

After the performance of DBN was verified, major concerns may arise on how it could practically contribute to the infectious diseases surveillance. Since disease forecasting is one of the core contents in surveillance, we further carried out Simulation 2, which mainly focused on how the DBN could improve the results of disease forecasting. Thus, the aim of Simulation 2 was to compare forecasting results obtained with and without the help of DBN. Specifically, two modelling strategies were separately employed: one was to use DBN to identify the risk factor(s) of HFMD before building the forecasting model, and the other one was to directly build the forecasting model without the help of DBN. The process and results of Simulation 2 were given as below.

### Simulation design

In Simulation 2, in order to enhance the representativeness of this study, we used another prototype study^18^, which involved the weekly childhood HFMD incidence and diurnal temperature range (DTR) data from 2011 to 2015 in Sichuan province, China. For all the scenarios considered previously in Simulation 2, *nonlinearity* and *delayed effect* of the original data have already been revealed by the prototype study^18^ of Simulation 2, and the *sample size* (260 weeks) also met the aforementioned requirement (at least three years). Therefore, Simulation 2 would focus on the scenario of *nuisance variables*. Specifically, three independently distributed nuisance variables $\,{Z}_{1,t}^{\ast }$, ${Z}_{2,t}^{\ast }$ and ${Z}_{3,t}^{\ast }$ were added into the original data, in the same way as in Simulation 1. As a consequence, the simulation scenario included five variables in total, where two of them (*HFMD*_*t*_ and *DTR*_*t*_) came from real-world study, and the other three (${Z}_{1,t}^{\ast }$, ${Z}_{2,t}^{\ast }$ and ${Z}_{3,t}^{\ast }$) were randomly created nuisance variables. Under such a simulation scenario, 5000 replicates of time series data were generated, and the length of each time series was 260 (the same as the original real data).

### Performance measures

To compare the performance of strategies with and without the help of DBN, the average values of fitting and forecasting MAPE (mean absolute percentage error) for the 5000 replicates were used as performance measures. Then the comparison of forecasting with and without the help of DBN could be conducted as follows. For each replicate of time series, we split the data into the training set (*t* = 1,2,…,230) and testing set (*t* = 231,…,260), and used the first set for model fitting and the second set for forecasting. In order to make comparison, the same type of forecasting model (the VAR model) was used in both strategies.

### Results interpretation

**Figure 5:**
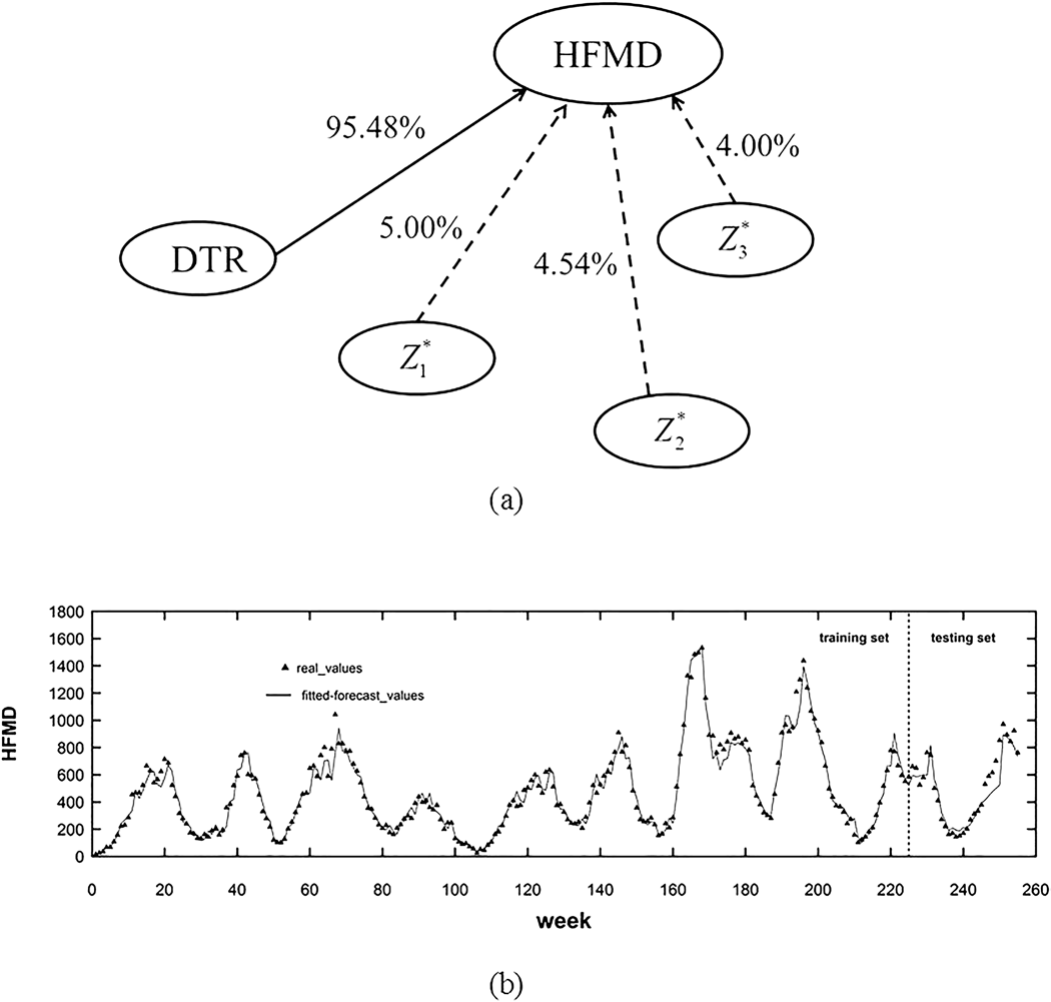
(**a**) The estimated DBN, where the solid lines represented the true positive rate (TPR), and dashed lines represented the false positive rate (FPR); (**b**) the time plots of the real values of HFMD time series (triangles) and results of the modelling strategy in combination with DBN (solid lines).

Figure 5 illustrated the main results of the modelling strategy with the help of DBN. First, it could be seen from Fig. 5(a) that among the 5000 replicates, the DBN could identify the real risk factor of *HFMD* (i.e., the *DTR*) with a true positive rate of 95.48% and false positive rates no more than 5%, which suggested that the forecasting model of HFMD only needed to take *DTR* into account. Then Fig. 5(b) showed the fitted and forecasted curves as well as the real-time series data of HFMD. It could be seen that both the fitted and forecasted values were close to the real ones, suggesting that the forecasting model had good fitting and forecasting performance with the help of DBN.

In addition, as presented in Table 3, while the fitting MAPEs of the two strategies (i.e., with or without DBN) were close to each other, the forecasting performance of the strategy with the help of DBN was better than that of the one without the help of DBN. To be more concrete, it could be seen that the DBN could improve the foresting results by reducing nearly 7% of the errors. This was reasonable since the nuisance variables, if entered into the forecasting model, would increase the complexity of model and made the forecasting results unstable and unreliable. Thus, the results indicated that by making the forecasting model parsimonious and efficient, the DBN could improve the ability of infectious diseases forecasting.

**Table 3:** The comparison of the two strategies*.

Strategy	Average Fitting MAPE	Average Forecasting MAPE
Strategy with DBN	10.7371%	15.0701%
Strategy without DBN	11.4175%	21.9365%

*The average fitting/forecasting MAPE was calculated as the mean value of the fitting/forecasting MAPEs through the 5000 replicates.

## Discussion

This paper proposed the DBN to identify the dynamic relations among infectious diseases surveillance data. It revealed that the DBN was competitive and even superior in relation to the Granger causality test and LASSO method under various scenarios (i.e., *sample size*, *mechanism* and existence of *nuisance variables*). In addition, we also found that sample size was important in identifying the dynamic relations among multiple variables. It was recommended that at least three years of weekly historical data were needed to guarantee the quality of infectious diseases surveillance. Besides, DBN also showed its potential value in infectious diseases surveillance by reducing the errors of forecasted incidences in the simulation study. Therefore, to our knowledge, this study contributed to infectious diseases surveillance in at least three ways.

1. This study utilized simulation designs to verify the performance of DBN in infectious diseases surveillance. The simulation design had two advantages. ① It could consider different scenarios of infectious diseases surveillance to make a relatively overall evaluation about DBN performance. Although some of the scenarios have already be considered to some extent by previous researches^11^, this work simultaneously explored all of them in context of infectious diseases surveillance. ② Simulation design guaranteed the practical meaning of study. In order to make simulation design close to the real-world situation as much as possible, this study used some previous real-world researches as prototypes to set simulation scenarios. Due to the closeness of simulation scenarios and real-world situation, the results of simulation study may provide reference for real-world study. As a result, simulation design in this study could serve as a bridge to apply theoretical findings of DBN to the practice of infectious diseases surveillance.
2. The results of this study showed that DBN had less FPRs than Granger causality test and LASSO method, especially when the sample size was large (*n* = 340). There are two possible reasons that can explain the better performance of DBN in comparison with the other two models. One reason is that both the DBN and LASSO involve shrinkage strategy that can help to reduce the FPRs by eliminating some trivial coefficients, but Granger causality test does not have such a shrinkage strategy. The other reason has something to do with the times of model building. To identify the dynamic relations among *p* variables, DBN only needs one time of model building (i.e., the vector autoregressive model), LASSO needs *p* times (at each time, one variable is set to be response variable while others the independent variables), and the pair-wise bivariate Granger causality test needs ${C}_{p}^{2}$ = *p*(*p*-1)/2 times. More times of modelling may lead to larger FPRs because of multiple comparisons. Although some compensation techniques (i.e., local FDR and *L*_1_ norm penalty) were used in this study, their contributions to reducing FPRs need more specific verification in the future.
3. This study implied how the DBN could help to improve the forecasting of infectious diseases. In summary, as shown in this study, the DBN could accurately and efficiently identify the relations among infectious disease and a variety of exogenous variables, especially in context of complicated data structures. This could make a real-world contribution by providing the Centers for Disease Control and Prevention (CDC) with the information of selecting prominent influencing factors of current infectious disease, which is extremely useful for building sophisticated deep-learning models to predict the start, peak and intensity of outbreak of infectious diseases in advance.

Although there were some interesting findings in this study, some limitations should also be acknowledged. First, our study only mentioned the forecasting as an example of how DBN could benefit the infectious diseases surveillance. However, the realistic work of infectious diseases surveillance is more comprehensive, which includes not only forecasting but also many other tasks such as early warning^34^ and intervention assessment^35^. Secondly, the challenges of infectious diseases surveillance need to be explored in deeper ways. For example, more forms of *nonlinearity* and *nuisance variables* should be considered. Another example is the challenge from the unobserved data or latent variable, since it is reasonable to believe that traditional analysis methods would be misleading and inappropriate when some of the important risk factors are unobserved in the surveillance data system^36,37^. To this end, we expect this work will contribute to further developments of infectious diseases surveillance.

### Ethical approval

Ethical approval was not required since the incidence rates used in this study are simulated data of infectious disease.

## Data Availability

The details of the data sets used in this paper have been described in the *simulation settings* part of the manuscript.
